# A Cost-Minimization Analysis of Tissue-Engineered Constructs for Corneal Endothelial Transplantation

**DOI:** 10.1371/journal.pone.0100563

**Published:** 2014-06-20

**Authors:** Tien-En Tan, Gary S. L. Peh, Benjamin L. George, Howard Y. Cajucom-Uy, Di Dong, Eric A. Finkelstein, Jodhbir S. Mehta

**Affiliations:** 1 Yong Loo Lin School of Medicine, National University of Singapore, Singapore; 2 Singapore National Eye Centre, Singapore; 3 Tissue Engineering and Stem Cell Group, Singapore Eye Research Institute, Singapore; 4 Singapore Eye Bank, Singapore; 5 Health Services and Systems Research, Duke-NUS Graduate Medical School, Singapore; 6 Lien Centre for Palliative Care, Singapore; 7 Department of Clinical Sciences, Duke-NUS Graduate Medical School, Singapore; Bascom Palmer Eye Institute, University of Miami School of Medicine, United States of America

## Abstract

Corneal endothelial transplantation or endothelial keratoplasty has become the preferred choice of transplantation for patients with corneal blindness due to endothelial dysfunction. Currently, there is a worldwide shortage of transplantable tissue, and demand is expected to increase further with aging populations. Tissue-engineered alternatives are being developed, and are likely to be available soon. However, the cost of these constructs may impair their widespread use. A cost-minimization analysis comparing tissue-engineered constructs to donor tissue procured from eye banks for endothelial keratoplasty was performed. Both initial investment costs and recurring costs were considered in the analysis to arrive at a final tissue cost per transplant. The clinical outcomes of endothelial keratoplasty with tissue-engineered constructs and with donor tissue procured from eye banks were assumed to be equivalent. One-way and probabilistic sensitivity analyses were performed to simulate various possible scenarios, and to determine the robustness of the results. A tissue engineering strategy was cheaper in both investment cost and recurring cost. Tissue-engineered constructs for endothelial keratoplasty could be produced at a cost of US$880 per transplant. In contrast, utilizing donor tissue procured from eye banks for endothelial keratoplasty required US$3,710 per transplant. Sensitivity analyses performed further support the results of this cost-minimization analysis across a wide range of possible scenarios. The use of tissue-engineered constructs for endothelial keratoplasty could potentially increase the supply of transplantable tissue and bring the costs of corneal endothelial transplantation down, making this intervention accessible to a larger group of patients. Tissue-engineering strategies for corneal epithelial constructs or other tissue types, such as pancreatic islet cells, should also be subject to similar pharmacoeconomic analyses.

## Introduction

The cornea is a five-layered structure in the anterior segment of the eye. The corneal endothelium pumps fluid out of the cornea, maintaining corneal deturgescence, which is crucial for corneal transparency and clear vision. Corneal endothelial dysfunction may be inherited e.g. Fuchs' endothelial dystrophy, or acquired due to surgical trauma e.g. pseudophakic bullous keratopathy. These are the leading causes of corneal transplantation in most developed countries [1].

The field of corneal transplantation has evolved rapidly in the past 10 years. Full-thickness penetrating keratoplasty (PK) techniques have been replaced by newer partial-thickness techniques for many corneal diseases [2]–[6]. In particular, endothelial keratoplasty (EK) techniques like Descemet's stripping endothelial keratoplasty (DSEK) and Descemet's membrane endothelial keratoplasty (DMEK) have been very successful for treating endothelial disease [7], [8]. Since 2005, the number of PK procedures performed in the USA has steadily dropped, while EK procedures have consistently risen, so much so that EK became the dominant procedure in 2012 [9]. DSEK involves transplanting a posterior lamellar corneal graft, consisting of donor corneal endothelium, Descemet's membrane, and a layer of posterior stroma, to replace dysfunctional recipient corneal endothelium. DSEK provides faster visual recovery, greater tectonic stability, less induced astigmatism, and lower rates of immunologic rejection, with comparable 3-year graft survival and endothelial cell loss rates to PK [5], [6], [10]. DSEK has also been shown to be more cost-effective than PK [11]. Furthermore, DSEK grafts can be precut prior to surgery, which simplifies the procedure and reduces operating time [12], [13].

However, EK, like any transplant procedure, is reliant on the availability of donor tissue [11]. Stricter tissue testing regulations and precautions against transmission of infectious disease have led to more costly processing and higher tissue discard rates, which both contribute to the rising cost of donor corneal tissue from eye banks [14], [15]. With aging populations and higher incidence of age-related corneal disease, the demand for donor tissue is also likely to increase [3], [16], [17]. Meanwhile, supply of donor tissue is unlikely to keep up, as most eye banks do not retrieve corneal tissue from donors above 75 years of age [18]. Stricter age criteria on donor tissue imposed by surgeons could dramatically worsen this situation further [18], [19]. Aging populations are therefore expected to worsen the worldwide shortage of transplantable corneal tissue [3]. Particularly in Asia, where donor retrieval rates are lower than the USA, this shortage is likely to be more acute. Such projections have engendered significant interest in alternative sources of transplantable corneal tissue [3], [16].

Tissue engineering is the use of cells and materials to produce functional substitutes for damaged tissue or organs [20]. Application of tissue engineering for the corneal endothelium is attractive for two main reasons: First, corneas are the most transplanted organ worldwide [4]. Second, tissue-engineered corneal epithelial constructs are already in clinical use [21], [22]. Tissue-engineered constructs comprising a monolayer of human corneal endothelial cells (HCECs), expanded within an *in vitro* environment, and seeded onto a membranous scaffold carrier should theoretically function as viable substitute graft material equivalent to precut EK donor tissues [23]. Recent advancements in reproducible culture of HCECs [3], [24]–[26] and improvements in surgical techniques like DSEK/DMEK [7], [8], [10], [27], [28] have made the clinical application of such tissue-engineered endothelial constructs a realistic possibility [29], [30]. In fact, clinical success of transplanted tissue-engineered endothelial constructs has already been demonstrated in a primate model [29], [30].

Performing EK with tissue-engineered endothelial constructs is within reach. However, whether or not it is economically viable remains an open question. Cost-minimization analysis is a form of pharmacoeconomic analysis that measures and compares the costs of two competing approaches that are assumed to provide equivalent outcomes [31]. This paper uses cost-minimization analysis to assess the economic feasibility of performing EK with tissue-engineered constructs, from the perspective of an ophthalmic institution in Singapore that possesses the surgical expertise to perform EK, but is lacking established infrastructure for the acquisition of transplantable EK tissue. For such an institution, would it be more prudent to: (a) invest in a laboratory to produce tissue-engineered EK grafts, or (b) utilize donor corneal tissue procured from eye banks for EK?

## Materials and Methods

A cost-minimization analysis comparing the use of tissue-engineered constructs versus procured tissue for EK was performed, based on the following assumptions:

1. EK procedures performed with either tissue-engineered constructs or precut procured tissue are identical in surgical technique, visual outcomes, complications and utility.
2. The institution possesses the surgical expertise, operating theatres and equipment for EK. However, said institution does not possess infrastructure for the acquisition of transplantable tissue. Therefore, tissue must either come from tissue engineering, or be procured from eye banks.
3. The institution does not possess an Automated Lamellar Therapeutic Keratoplasty (ALTK) system (Moria, Antony, France) for cutting EK grafts in operating theatres. Therefore, any donor corneas procured from eye banks must be precut before use in surgery.
4. Obtaining transplantable EK tissue by the tissue-engineering strategy requires the set-up of a laboratory capable of producing tissue-engineered EK grafts (henceforth referred to as the “laboratory”), while the procured-tissue strategy requires a facility capable of receiving, storing and precutting donor tissue from eye banks (henceforth called the “facility”).
5. The laboratory/facility will be nested within the institution, and will have access to a surgical sterilization unit. Therefore, these costs can be excluded.
6. The amount of physical space and renovations required for either the laboratory or the facility will be similar, and these expenses can therefore be excluded.
7. Utilities costs for both the laboratory and facility will be similar, and can therefore be excluded.
8. All corneal tissue, whether used for tissue culture or for precutting, is acquired from the same source: Florida Lions Eye Bank, Miami, FL, USA.
9. All corneas arrive in matched pairs from the same donor. Therefore, corneas of a pair share delivery courier costs, and can also be handled and precut with the same set of sterile instruments, without sterilization in between.
10. There will be no tissue wastage; all corneas arrive on time, and all precutting, isolation and culture procedures will be successful. The costs of Quality Assurance (QA) testing for both tissue-engineered constructs and precut donor tissue will be included, but all will be of sufficient quality for transplant.

The authors are based in Singapore, and therefore this model was based on an institution in Singapore. All quotations were obtained in Singapore Dollars (S$), and subsequently converted to United States Dollars (US$), with S$1 equivalent to US$0.797, according to the international exchange rate on 17 June 2013.

### Cost of Tissue-Engineered Constructs

**Figure 1:**
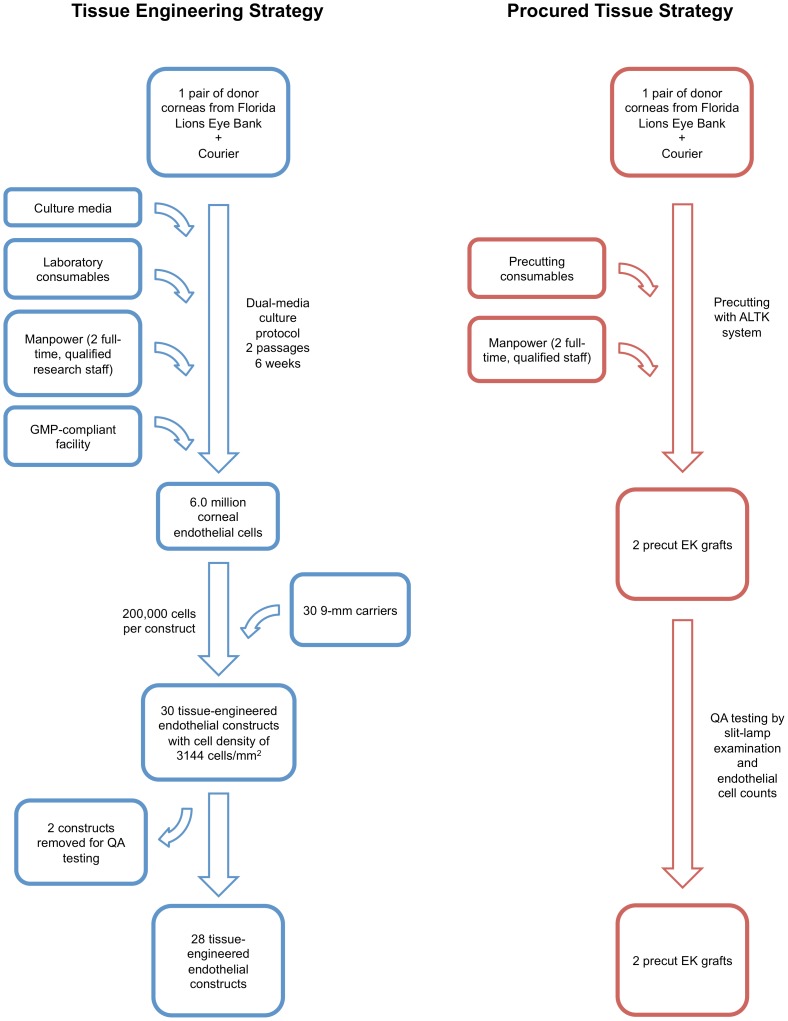
Overview of transplant strategies. Overview of the tissue engineering strategy (in blue) and the procured tissue strategy (in red). Abbreviations: GMP, Good Manufacturing Practice; QA, Quality Assurance; ALTK, Automated Lamellar Therapeutic Keratoplasty; EK, endothelial keratoplasty.

Cost calculations were based on a published isolation and culture protocol that is able to reliably expand HCECs up to the third passage [25], [26]. An overview of this process is illustrated in Figure 1. Briefly, HCECs were isolated from a pair of transplant-grade donor corneas by a two-step “peel-and-digest” method and were expanded using a dual-media culture system [25], [26]. However, HCECs from different donors exhibit marked donor-to-donor variability [24], [32]. Therefore, a more conservative estimate was used for calculations: HCECs were used at the end of the second passage (P2), ensuring that all batches maintain good morphology. Using this protocol, HCECs from one pair of corneas can be expanded to 6.0 million cells on average by the end of P2, in 6 weeks [25]. At the end of P2, 200,000 HCECs were seeded onto each 9 mm-diameter plastic compressed collagen carrier, producing 30 tissue-engineered constructs, each with a projected endothelial cell density (ECD) of 3144 cells/mm^2^. From each batch of 30 constructs, 2 were selected randomly for QA testing, which involved testing for mycoplasma, endotoxin, and corneal endothelium-associated markers including Na^+^/K^+^-ATPase, ZO-1, GPC-4, CD200, SLC4A11, COL8A1/2, and CYYR1 [33], [34]. These constructs used for QA testing were not transplanted.

It was assumed that one pair of corneas arrived every fortnight, and took 6 weeks to culture. Pairs of corneas at different stages of the protocol could be cultured simultaneously. Therefore, every fortnight, one batch of 28 transplantable constructs was ready, and one new pair of corneas arrived. At any point in time, there were 3 batches being processed concurrently, at different stages. This produced an average of 14 constructs per week. Two qualified, full-time staff should be able to manage this workload.

The monetary costs involved in setting up and running a laboratory for the implementation of this protocol were derived in consultation with senior scientists from the Tissue Engineering and Stem Cell Group of the Singapore Eye Research Institute, who have developed this protocol. Conceptually, costs were divided into investment costs and recurring costs. Investment costs were the capital outlay for the necessary equipment (Table 1). Recurring costs were calculated on a per-pair-of-corneas basis, and included costs of manpower, donor corneas for culture (inclusive of door-to-door courier service from the eye bank), culture media components, laboratory consumables, plastic collagen compressed carriers, QA testing, and rental of a suitable laboratory compliant with Good Manufacturing Practice (GMP) standards. Rental cost of a GMP-compliant laboratory was estimated in consultation with the director of such a laboratory in Singapore. Annual recurring costs were then derived by multiplying the recurring cost per pair of corneas by the number of cornea pairs processed in one year. Recurring costs are listed in Table 2. Applying an annual amortization rate of 20% to investment costs [35], and adding this to annual recurring costs derived total annual cost. Tissue cost per EK was calculated by dividing total annual cost by the total number of constructs produced per year, less those used for QA testing.

**Table 1:** Investment costs.

Tissue-engineered constructs			Procured donor tissue		
Item	No.	Unit cost (US$)	Item	No.	Unit cost (US$)
4°C laboratory refrigerator	1	3,188	Eye bank refrigerator (4–8°C, with temperature-recording graph)	1	7,500
−20°C laboratory freezer	1	3,985	Specular microscope	1	40,000
Dissection microscope	1	7,970	Slit-lamp biomicroscope	1	7,000
Inverted light microscope	1	3,188	ALTK system	1	100,000
Biosafety cabinet	1	6,376	Ultrasound pachymeter	1	7,000
CO_2_ incubator	1	4,782	Laminar flow hood	1	7,000
Centrifuge	1	9,564	Small box freezer	1	250
Vacuum pump	1	957			
Single channel pipettes	1	797			
Serological pipet-aid	1	399			
Forceps	1	168			
Hemocytometer	1	399			
Water bath	1	957			
Water purification system	1	7,970			
Investment cost (US$)		50,700	Investment cost (US$)		168,750

Abbreviations: ALTK system, Automated Lamellar Therapeutic Keratoplasty system (Moria, Antony, France).

**Table 2:** Recurring costs.

Tissue-engineered constructs		Procured donor tissue	
Item	Cost per pair of corneas (US$)	Item	Cost per pair of corneas (US$)
1 pair of donor corneas	2,900×2	1 pair of donor corneas	2,900×2
Courier	250	Courier	250
Manpower	5978	Manpower	770
Culture media components	538	Precutting consumables	250×2
Laboratory consumables	54		
Plastic compressed collagen carriers	200×30		
QA testing	66		
Rental of GMP-compliant laboratory	5,579		
**Recurring cost per pair of corneas (US$)**	**24,265**	**Recurring cost per pair of corneas (US$)**	**7,320**
No. of cornea pairs per year	26	No. of cornea pairs per year	364
**Annual recurring cost (US$)**	**630,890**	**Annual recurring cost (US$)**	**2,664,480**

Abbreviations: QA, Quality Assurance; GMP, Good Manufacturing Practice.

### Cost of Utilizing Procured Corneal Tissue

Utilization of procured corneal tissue for EK requires a facility, which is able to perform precutting of donor corneal tissue, and run QA testing by slit-lamp examination and endothelial cell counts. An overview of this process can be found in Figure 1. To allow comparison with the tissue engineering strategy, it was assumed that this facility produced 14 precut EK grafts a week. Therefore, 7 pairs of corneas were processed per week. Two qualified, full-time staff, using 1 ALTK system, should be able to manage this workload.

Monetary costs involved in the setup and running of such a facility were calculated in consultation with the manager of an established local eye bank. These were similarly divided into investment costs (Table 1) and recurring costs (Table 2). Recurring costs were first calculated on a per-pair-of-corneas basis, and included costs of manpower, donor corneas (inclusive of door-to-door courier service) and precutting consumables. Annual recurring costs were then derived by multiplying the recurring cost per pair of corneas by the number of cornea pairs processed in one year. Applying an annual amortization rate of 20% to investment costs [35], and adding this to annual recurring costs derived total annual cost. Tissue cost per transplant was calculated by dividing total annual cost by the total number of precut grafts produced per year.

### Sensitivity Analysis

**Figure 2:**
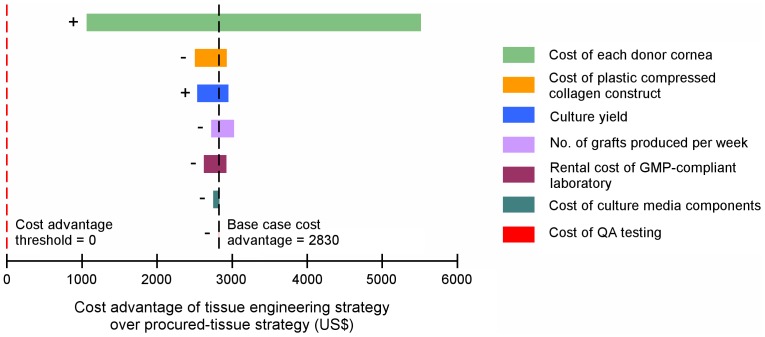
One-way sensitivity analysis. One-way sensitivity analysis of selected variables on the cost advantage of the tissue engineering strategy over the procured-tissue strategy. + indicates that the cost advantage increases as that variable increases, while - indicates that the cost advantage decreases as that variable increases. Abbreviations: GMP, Good Manufacturing Practice; QA, Quality Assurance.

A number of assumptions were made in the cost-minimization analysis. In view of this, various types of sensitivity analyses were performed to test the robustness of the results. Key inputs of the model were identified (Table 3), and varied in order to examine their effect on the overall outcome of the cost-minimization analysis. Each input variable was assigned a reasonable sensitivity range (Table 3). Where possible, these sensitivity ranges were guided by available data. Otherwise, half the base case value was taken as the lower limit, and double the base case value was taken as the upper limit. One-way sensitivity analyses were performed in which each of the seven variables was varied individually, and the outcome examined (Figure 2). The outcome measured in this analysis was the cost advantage of the tissue engineering strategy over the procured-tissue strategy. Cost advantage was derived by subtracting tissue cost per transplant of the tissue engineering strategy from tissue cost per transplant of the procured-tissue strategy.

**Table 3:** Sensitivity ranges.

Variable	Base case value	Sensitivity range	Remarks
Cost of each donor cornea (US$)	2,900	1,000–5,800	2,900 is the cost of a transplant-grade donor cornea from Florida Lions Eye Bank, Miami, FL, USA. 1,000 is the cost of a transplant-grade donor cornea from the National Eye Bank of Sri Lanka, Colombo, Sri Lanka.
Cost of plastic compressed collagen construct (US$)	200	100–500	
Rental of GMP-compliant laboratory per pair of corneas (US$)	5,579	2,789.5–11,158	
Cost of culture media components per pair of corneas (US$)	538	269–2,690	The GMP-compliant equivalents of some culture media components can be 5 times more expensive than their non-GMP-compliant counterparts. Therefore, the upper limit of the sensitivity range was set at 5 times the base case value.
Cost of QA testing per pair of corneas (US$)	66	33–132	
Culture yield (no. of constructs produced per pair of corneas, before QA testing)	30	15–60	
No. of transplantable EK grafts produced per week (for both strategies)	14	7–28	

Abbreviations: GMP, Good Manufacturing Practice; QA, quality assurance; EK, endothelial keratoplasty.

Probabilistic sensitivity analyses were also performed, in which all seven variables were varied simultaneously. Each variable was assumed to follow a triangular distribution within its designated sensitivity range (Table 3), and a specific value chosen by random sampling. These values were used to calculate the tissue cost per transplant for both strategies, which were then compared. This simulation was run 10,000 times, in order to determine which strategy produced transplantable tissue at a lower cost in the majority of simulations.

Additionally, in order to make the results of this study more generalizable, two alternative specific real-world scenarios were tested. First, some eye banks are able to prepare EK grafts with a DMEK technique [8], [9], [36]. If the facility were to prepare procured donor tissue with such a technique, they would not need to invest in an ALTK system or require precutting consumables. As an ALTK system requires a hefty investment (US$100,000), this scenario was specifically simulated and compared against the tissue engineering strategy. Second, many eye banks in the United States of America (USA) are able to precut EK grafts before distribution [9], [12], [13], [19]. Florida Lions Eye Bank provides precut EK grafts for US$3,400 (as opposed to US$2,900 for non-precut tissue). As a result, many corneal surgeons in the USA have precut EK grafts delivered to the operating theatre just prior to surgery. In so doing, they obviate the need for precutting equipment (including an ALTK system) and consumables, QA testing, or manpower for a dedicated facility. Their recurring costs would only include that of the precut tissue and courier service, and they would only need to invest in an eye bank refrigerator for short-term storage of procured precut EK grafts. As this is a common occurrence in the USA, specific cost calculations were also performed for this scenario and compared with the tissue engineering strategy.

## Results

### Investment Costs

In the tissue engineering strategy, the investment cost for the laboratory was US$50,700 (Table 1). In contrast, with the procured-tissue strategy, the investment cost needed for the facility was US$168,750 (Table 1). A large proportion of this figure came from the US$100,000 cost of the ALTK system used for precutting grafts.

### Recurring Costs

In order to produce 14 tissue-engineered constructs per week, the laboratory processed 1 pair of donor corneas every 2 weeks. The recurring cost of processing each pair was US$24,270. In 1 year, the laboratory operating at this rate processed 26 pairs of corneas, producing 728 transplantable constructs. Therefore, the annual recurring cost for the laboratory was US$630,890 (Table 2).

In contrast, the facility processed 7 pairs of donor corneas every week to produce 14 precut grafts per week. The recurring cost of processing each pair was US$7,320. At this rate, the facility processed 364 pairs of corneas in 1 year to produce 728 precut grafts. The annual recurring cost for the facility was US$2,664,480 (Table 2).

### Tissue Cost per Transplant

Producing 728 transplantable constructs per year, the laboratory required a total annual cost of US$641,030. Therefore, the laboratory produced transplantable tissue for US$880 per transplant on average (Table 4). At the same rate of production, the facility required a total annual cost of US$2,698,230. Therefore, the facility produced transplantable tissue for US$3,710 per transplant (Table 4).

**Table 4:** Tissue cost per transplant.

	Tissue-engineered constructs	Procured donor tissue
Investment cost (US$)	50,700	168,750
Amortization of investment cost (20%) (US$)	10,140	33,750
Annual recurring cost (US$)	630,890	2,664,480
Total annual cost (US$)	641,030	2,698,230
No. of transplants per year	728	728
**Tissue cost per transplant (US$)**	**880**	**3,710**

Abbreviations: None.

### Sensitivity Analysis

Sensitivity analyses performed support the outcome of the cost-minimization analysis. In one-way sensitivity analyses, varying individual variables (Table 3) in the model still resulted in the same conclusion. Within the assigned sensitivity ranges, the tissue engineering strategy was always the preferred strategy; cost advantage never crossed the threshold of 0 (Figure 2). As an illustration, if one were to ignore the assigned sensitivity ranges, the cost of the plastic compressed collagen carrier would have had to be multiplied 14 times in order for both strategies to be equal in cost. The equivalent multipliers for rental cost of a GMP-compliant laboratory, cost of culture media components and QA testing cost were 15, 150 and 1200 respectively. With the remaining three variables, there was no positive value at which cost advantage reached 0. Two of the variables (cost of donor cornea and culture yield) were positively related to the cost advantage (labeled “+” in Figure 2); as these variables increased, the cost advantage of the tissue engineering strategy increased. The other five variables were inversely related to the cost advantage (labeled “−“). In terms of the magnitude of effect, it was found that the largest determinant of the cost advantage was the cost of each donor cornea. This was followed by the cost of the plastic compressed collagen carrier, and by the culture yield from each pair of corneas.

In probabilistic sensitivity analyses, all seven variables were varied simultaneously across 10,000 simulations. The tissue engineering strategy produced transplantable tissue at a lower cost than the procured-tissue strategy in 100% of simulations.

As an additional form of sensitivity analysis, two specific alternative scenarios were tested. First, if the facility in the procured-tissue strategy were to prepare EK grafts with a DMEK technique (without the need for an ALTK system or precutting consumables), the tissue cost per transplant would be reduced to US$3,430. Second, if eye surgeons were to accept delivery of precut EK grafts direct from the eye bank, as is the case for many corneal surgeons in the USA, the tissue cost per transplant would be US$3,660. Both of these figures are still significantly more than the US$880 per transplant produced by the tissue engineering strategy.

## Discussion

The technique of cultivating HCECs from one donor *ex vivo* and transplanting them on a carrier to treat endothelial disease in the recipient was conceptualized over 30 years ago [23]. Since then, however, efforts to translate this into clinical practice have been hampered by difficulty in culturing HCECs and the lack of effective surgical techniques to transplant them. Recently though, greater understanding in the cell biology of HCECs [37]–[40] has led to the establishment of reliable HCEC culture protocols [3], [24]–[26]. HCECs can now be consistently expanded up to the third passage, while retaining their unique cellular morphology and the expression of characteristic markers indicative of the corneal endothelium [24], [25]. Suitable carriers for these cultured HCECs have also been successfully tested [41]–[45]. Finally, the advent and success of EK techniques like DSEK/DMEK enables the effective surgical delivery of these constructs [7], [8], [10], [27], [28]. Such recent advances may have made tissue-engineered endothelial constructs a realistic prospect, but can they be produced at a competitive cost?

The results of this cost-minimization analysis indicate that tissue engineering can produce transplantable corneal endothelial tissue at a fraction of current costs (Table 4). Both investment costs and recurring costs were lower for tissue-engineered constructs compared to tissue procured from eye banks. Although certain assumptions were necessary to allow comparison between the two strategies, we performed multiple sensitivity analyses to attempt to account for uncertainty in our estimates, and variation in costs across different settings or countries. These sensitivity analyses performed demonstrate that the results of this study are robust and may even be generalizable across various settings. In particular, even if the substantial cost of the ALTK system was removed from the procured-tissue strategy, or if one were to procure already-precut tissue from eye banks (as is common practice in the USA), the tissue engineering strategy still provides a significant cost advantage. This reduction in the cost of transplantable tissue could potentially make corneal endothelial transplants more accessible for a large number of patients. Furthermore, one-way sensitivity analyses have identified two key variables that are positively related to the cost advantage of the tissue engineering strategy over procured corneal tissue: the cost of donor corneas, and the culture yield (indicated “+” in Figure 2). If the costs of donor corneal tissue from eye banks continue to rise (as they have done historically) [14], and as isolation and culture protocols for HCECs are refined and culture yield improves, the cost of corneal endothelial transplantation could fall even further.

A key advantage of the tissue engineering strategy is that *in vitro* expansion of HCECs allows tissue from one donor to treat multiple recipients [3], [41]. Widespread adoption of this strategy should increase the overall supply of transplantable corneal endothelial tissue, and help to meet the current and expected future shortfalls in tissue for corneal endothelial transplants [3]. In our center, and in many others in Asia, demand for corneal endothelial transplants exceeds supply. There are always patients on the waiting list, and donor tissue is not always readily available. However, if the supply of donor tissue was more predictable, like in the tissue engineering strategy, patients on the waiting list could be scheduled for surgery based on when the grafts will be ready, reducing waiting time for patients, and minimizing week-to-week fluctuation in transplant numbers. Also, because multiple transplants can be performed from one donor cornea, serological and other Quality Assurance (QA) tests need only be done on the one source cornea, resulting in significant cost savings, and further reducing delays in transplantation.

The implications of this analysis are not limited to corneal endothelial transplantation alone. This analytic model could potentially be applied to other fields, including: other types of tissue-engineered constructs e.g. epithelial constructs for ocular surface reconstruction, as well as transplantation of other tissues like pancreatic islets for the treatment of Type 1 Diabetes Mellitus [46], [47]. Stem cell-based alternatives to donor pancreatic islet tissue are currently an area of active research [48]–[53]. As cell culture protocols and techniques become more clearly defined, it would be important to conduct similar pharmacoeconomic analyses of these stem cell-based strategies for pancreatic islet transplantation as well. To the best of our knowledge, no such studies have been attempted yet.

The results of this study should be interpreted with a measure of caution. Cost-minimization analysis is grounded in the assumption that the competing therapies produce equivalent outcomes [31]. While tissue-engineered endothelial constructs should, in theory, perform as well as precut donor tissue in terms of surgical ease, complication rates and outcomes, this has yet to be proven. We do not know if the two approaches will have equivalent success rates, long-term graft survival or quality of life after transplantation. There is currently no data from human studies that proves their therapeutic equivalence, and future clinical trials in this area are needed. Our pharmacoeconomic analyses are complementary to clinical trials in this area, and should be interpreted alongside such work. If data arises showing that the two therapies do indeed differ in outcome, then a full cost-effectiveness analysis will be necessary. Nevertheless, the authors feel that for the purposes of this analysis, this assumption is reasonable. From a surgical perspective at least, corneal endothelial transplantation with tissue-engineered grafts is technically feasible, and should not be significantly different from precut EK grafts in terms of surgical ease or complication rates. Levis et al found that their plastic compressed collagen carrier could be successfully manipulated and deployed using current graft delivery devices without complication [41]. Also, cultured HCECs express cellular markers characteristic of *in vivo* HCECs, such as Na^+^/K^+^-ATPase, that are in part responsible for the function of the corneal endothelium in maintenance of corneal clarity [24]. Therefore, although more clinical research is needed in this field, the authors feel that the above assumptions are not unreasonable.

Some corneal transplant surgeons have reported using single donor corneas for both deep anterior lamellar keratoplasty (DALK) and EK procedures in two separate recipients [54]–[58]. This technique of split cornea transplantation, if widely adopted by surgeons or eye banks, could potentially help to alleviate the shortage of donor corneal tissue. Also, the use of a single donor cornea for two separate transplants would significantly reduce the cost of procured donor tissue for each transplant procedure. However, the impact of this concept is limited by the fact that one of the two procedures would have to be a DALK, which is less commonly performed than EK procedures like DSEK. Even though DALK is performed quite often in Singapore [59], it only accounts for 1.89% of corneal transplant procedures performed in the United States [9]. Furthermore, DALK is often performed with tissue that has endothelium that would be unsuitable for EK surgery [60] and hence, this is unlikely to have significant impact on the results of this cost-minimization analysis.

If tissue-engineered endothelial constructs are to be produced on a large scale, the validity of the QA tests used must be well established. Presently, there are a few markers specific to HCECs that can be tested for, such as Na^+^/K^+^-ATPase, ZO-1, GPC-4, CD200, SLC4A11, COL8A1/2, and CYYR1 [33], [34]. However, none of these have been directly linked to clinical success of the transplant. Corneal epithelial cultures, in contrast, have a validated QA test using expression of ΔNp63α that has been directly linked to successful clinical outcomes [22]. Ideally, a similarly validated QA test should be developed for tissue-engineered endothelial constructs before use becomes widespread.

Finally, there are limits to the generalization of these results. This analysis was performed in the authors' setting of Singapore. While we have performed different sensitivity analyses in an attempt to make these results generalizable across various settings, there are certain scenarios that are beyond the scope of this paper. First, this analysis was designed from the perspective of an institution that currently has no means of acquiring EK tissue. Therefore, the results are not immediately applicable to an institution that has already made investments in an eye bank and/or an ALTK system. Also, there are many considerations unique to healthcare institutions in the developing world, and various cost factors may be quite different. Therefore, these results may not be directly applicable to institutions in the developing world. In addition, we acknowledge that institutions in different countries operate within vastly different healthcare settings. Our analysis simply shows that a tissue engineering strategy can potentially increase the supply of transplantable tissue, which will in turn lower the cost of tissue per corneal endothelial transplant. Whether these cost savings will be passed on to patients or enjoyed as additional revenue for the institution will vary based on market characteristics. There are many economic factors besides the production cost of a therapy for a particular institution to consider in its investment decisions, such as government reimbursement or subsidy rates. More detailed market and budget impact analyses would have to be performed by each institution, taking into account the unique healthcare environment they operate in. Such issues are beyond the scope of this paper.

In conclusion, tissue-engineered constructs for corneal endothelial transplantation can be produced at a lower cost than donor tissue procured from eye banks. Future clinical trials are needed to establish the therapeutic equivalence of both these tissue sources for EK. Tissue-engineered endothelial constructs have the potential to increase the supply of transplantable corneal endothelial tissue and bring the overall costs of corneal endothelial transplantation down.
